# Role of Galectins in Tumors and in Clinical Immunotherapy

**DOI:** 10.3390/ijms19020430

**Published:** 2018-02-01

**Authors:** Feng-Cheng Chou, Heng-Yi Chen, Chih-Chi Kuo, Huey-Kang Sytwu

**Affiliations:** 1 Department and Graduate Institute of Microbiology and Immunology, National Defense Medical Center, Taipei 114, Taiwan; fengchengchou@gmail.com; 2 Graduate Institute of Life Sciences, National Defense Medical Center, Taipei 114, Taiwan; vicky0128n@gmail.com; 3 Teaching and Research Office, Tri-Service General Hospital Songshan Branch, National Defense Medical Center, Taipei 105, Taiwan; vampirelydia@mail.ndmctsgh.edu.tw

**Keywords:** galectin-1, galectin-3, galectin-9, immunotherapy, galectin inhibitors

## Abstract

Galectins are glycan-binding proteins that contain one or two carbohydrate domains and mediate multiple biological functions. By analyzing clinical tumor samples, the abnormal expression of galectins is known to be linked to the development, progression and metastasis of cancers. Galectins also have diverse functions on different immune cells that either promote inflammation or dampen T cell-mediated immune responses, depending on cognate receptors on target cells. Thus, tumor-derived galectins can have bifunctional effects on tumor and immune cells. This review focuses on the biological effects of galectin-1, galectin-3 and galectin-9 in various cancers and discusses anticancer therapies that target these molecules.

## 1. Introduction

Galectins are a family of lectins composed of one or two carbohydrate-recognition domains (CRDs) that bind to beta-galactoside-containing glycans. To date, 15 galectins have been identified in mammals and 11 are found in humans, acting both intracellularly and extracellularly. Galectins can be classified into three groups based on their structure: (1) prototype galectins that contain one CRD that can form homodimers, including galectin-1, galectin-2, galectin-5, galectin-7, galectin-10, galectin-11 galectin-13, galectin-14 and galectin-15; (2) tandem repeat-type galectins that contain two CRDs and are connected by a flexible linker, including galectin-4, galectin-6, galectin-8, galectin-9 and galectin-12; and (3) chimeric-type galectin-3 that contains a CRD domain and an N-terminal extension that can form oligomers to increase their binding avidity (galectin-5, -11, -15 and -6 are not found in humans).

Galectins are soluble proteins that are widely expressed in various cell types and mediate their functions both intracellularly and extracellularly. Although galectins do not have any known signal sequence for their transport, it is likely to be a nonexocytotic pathway; for example, cells infected with the Epstein-Barr virus release galectin-9 via an exosome-mediated mechanism [1]. In general, the functions of galectins include the regulation of cell growth, apoptosis, pre-mRNA splicing, cell-cell and cell-matrix adhesion, cellular polarity, motility, differentiation, transformation, signal transduction and innate/adaptive immunity. Due to the diverse functions of galectins, such as in apoptosis, angiogenesis, cell migration and tumor-immune escape, altered levels have been implicated in cancer biology [2]. For example, in tumor cells, intracellular galectins can enhance oncogenic signals and reduce apoptosis that promote tumor transformation and proliferation (reviewed in [3]). By contrast, the extracellular galectins bind to cell surface glycoproteins and form galectin lattices, depending on the glycosylated sites. The galectin–receptor lattice can modulate the functions of the receptor and support either surface retention or transportation of the receptors [4].

The ligands of galectins are glycoproteins and glycolipids that contain different degrees of oligosaccharide modifications (*N*- and *O*-linked glycans). The selectivity and factors that influence how each galectin member binds to glycoproteins depend on the glycosylation sites (sequence-encoded information), on glycosylation levels in Golgi complex processing (depending on glycosyltransferase activity) and glycol-conjugate formations (e.g., LacNAc, 2-*O*-glycans, complex branched *N*-glycans or sialylated structures) [5,6]. Thus, given the preferred glycan branch of an individual galectin, each type can bind to a set of glycoconjugates on the cell surface that mediates specific functions. Interestingly, some galectins regulate innate and adaptive immune responses by binding to a panel of glycoproteins on immune cells. For example, galectin-1 binds to CD2, CD3, CD7, CD43 and CD45 on T cells, which downregulate immune responses by inducing apoptosis. By contrast, galectin-9 has a dual function, by binding to T cell immunoglobulin mucin 3 (TIM-3) expressed on T cells or dendritic cells, which induces apoptosis or inflammatory responses, respectively. In this review, we discuss the relationship between tumor-derived galectins and tumor prognosis. In addition, given the multiple immune regulatory functions of galectins on T cells, we also summarize the results of clinical trials that used galectin inhibitors combined with various forms of chemotherapy or immunotherapy.

## 2. Role of Galectins in Tumor Progression and Immune Surveillance

### 2.1. Human Galectins

Among the 11 galectins identified in humans, galectin-1, galectin-3 and galectin-9 have been the most extensively investigated in different fields including cell biology and immunology. Importantly, the roles of these three forms have been closely linked to cancer biology. The functions of these galectins in tumors include enhancing oncogenic signal pathways, regulating tumor cell growth or apoptosis, modulating cell migration and suppressing immune responses.

### 2.2. Correlation of Galectin-3 and/or Galectin-1 in Cancers

Galectin-3 and galectin-1 have been investigated extensively in various tumors [2]. Clinically, thyroid malignancies of epithelial origin display increased galectin-1 and galectin-3 expression compared with benign thyroid adenomas [7]. In human endometrial cancers, the expression of galectin-1 is upregulated in uterine adenocarcinomas compared with normal adjacent endometrium, whereas expression of galectin-3 is downregulated in endometrial cancer cells compared with normal mucosa. Interestingly, tumors with galectin-3 expressed in the cytoplasm were characterized by a deeper invasion of the myometrium compared with lesions where galectin-3 was found both in the nucleus and cytoplasm [8]. The localization of galectin-3 in normal cells and different stages of cancer cells were further investigated in cases of colorectal cancer progression. In line with previous findings, strong cytoplasmic expression of galectin-3 was associated with later phases of tumor progression and was inversely correlated with the survival of patients [9]. In cases of human bladder cancer, increased mRNA expression of galectin-1 in transitional-cell carcinomas was positively correlated with histological grade and clinical stage. However, the expression of galectin-3 only showed increases in carcinomas without being correlated with histological grade [10]. Then, the biological effects of galectins linked to tumors have been investigated. For example, increased levels of galectin-3 are involved in liver metastasis, venous invasion and lymph node metastasis of colorectal cancer [11]. Galectin-3 levels were also increased in the blood stream of cancer patients and this promoted cancer metastasis via binding to cell surface-associated mucin 1 (MUC1) on cancer cells, in turn leading to the exposure of smaller cell-surface adhesion molecules/ligands including CD44 and ligand(s) for E-selectin [12]. The interaction of galectin-3 with MUC1 has been reported to increase the association of MUC1 with the epidermal growth factor receptor (EGFR), which then promotes EGFR homo-/heterodimerization and subsequently increased and prolonged EGFR activation and signaling. These mechanisms might contribute to EGFR-associated tumorigenesis and cancer progression and could also influence the effectiveness of blocking the action of EGFR in patients undergoing cancer therapy [13]. Moreover, increased galectin-3 expression in gastric cancer cells contributes to cellular unresponsiveness to interferon gamma (IFN-γ) by facilitating the AKT/GSK-3β/SHP2 signaling cascade [14]. Importantly, several cytokines are also glycoproteins whose functions can be neutralized by extracellular galectins. One elegant study demonstrated that tumor-derived galectin-3 captured IFN-γ in tumor matrices and subsequently downregulated IFN-γ-induced chemokine gradients. Inhibition of galectin-3 in human tumor biopsies enhanced IFN-γ-induced CXCL-9 chemokine expression, suggesting that the blockade of galectin-3 in tumor cells may promote T cell tumor infiltration and activation [15].

### 2.3. Galectin-9 and Tumor Metastasis

Galectin-9 has attracted much attention because of its multiple biological functions and strong immunomodulatory effects. Expression of galectin-9 in solid tumors has been linked to tumor cell adhesion or metastasis. Thus, the galectin-9 expression level was correlated with cellular adhesion and aggregation in melanoma cells [16], oral squamous cell carcinomas [17], breast cancer [18] and hepatocellular carcinoma (HCC) [19]. These studies have demonstrated that the high expression level of galectin-9 in tumor cells promoted tumor cells aggregation in vitro and in mouse models, whereas downregulation of galectin-9 in these cells correlated with cell invasion. Additionally, the following studies further demonstrated that administration of galectin-9 in mice suppresses lung metastasis of melanoma cells via inhibition of the binding of adhesive molecules on tumor cells to ligands on vascular endothelium and extracellular matrix [20]. In clinical studies, an association of the expression level of galectin-9 with tumor metastasis has been established in breast cancer and in HCC. Patients with galectin-9 negative breast cancers showed high potential with distant metastases and correlated with higher histopathologic grades when compared with patients with galectin-9 positive breast cancer. In addition, the cumulative disease-free survival rate for galectin-9-positive patients was better than in a galectin-9-negative group [18]. Consistent with those findings, in patients with HCC, the decreased expression of galectin-9 was linked to lymph node metastasis, vascular invasion, intrahepatic metastasis and poor survival of patients [19]. In summary, the high expression level of galectin-9 in primary cancer lesions promoted cell-matrix interactions, and metastatic lesions displayed decreased galectin-9 expression, suggesting that galectin-9 might suppress tumor metastasis. Moreover, these results indicated that galectin-9 might serve as a prognostic factor with antimetastatic potential in patients with breast cancer and HCC; however, whether galectin-9 also has similar effects in other cancer types is still unclear. 

A detailed summary of the differential expression levels of galectins in various tumors has been provided in [2]. Intriguingly, the relationship between the expression level of galectins and the malignancy of a given tumor is context dependent, suggesting that many factors should be taken into consideration, such as tumor type and stages, and the involvement of different galectins. 

## 3. Galectins Are Involved in Immune Escape by Tumors

### 3.1. Galectin-1, -3 and -9 Regulate Immune Responses via Different Receptors

Despite the multiple functions of galectins in cancer cell growth, they also have strong immunomodulatory functions that regulate both innate and adaptive immunity by binding to surface glycoproteins. Tumor cells use various strategies to escape immune attack or even regulate immune responses. For example, galectin-1 and galectin-3 can induce T cell apoptosis by binding to CD45 and CD7 leukocyte proteins to induce apoptosis. Galectin-9, unlike other galectin members with many cellular receptors, binds specifically to the Tim-3 cell surface molecule on Th1 cells and induces apoptosis [6]. Ligands for galectin-1, 3 and 9 are listed in Table 1. Reports suggest that the expression of these galectins in tumor cells might help ablate the immune response to tumors.

### 3.2. Galectin-1 Modulates the Antitumor T Cell Response

There have been reports that tumor microenvironments display enhanced expression of galectins linked to tolerogenic status. Galectin-1 exhibits strong immunoregulatory functions which have been demonstrated elegantly in a mouse melanoma model. Targeted inhibition of galectin-1 is correlated with enhanced T cell-mediated tumor clearance, demonstrating a strong immunosuppressive effect on T cells [42]. In clinical studies, increased galectin-1 levels have been reported in leukemic tumors, such as neoplastic Reed-Sternberg cells in patients with classic Hodgkin lymphomas [43] and in those with leukemic cutaneous T-cell lymphomas [44]. In those studies, increased galectin-1 promoted Th2 responses or expansion of Treg cells and inhibited the proliferation of other T cell types, suggesting that blockage of galectin-1 might enhance antitumor activity. In solid tumors, expression of galectin-1 was inversely correlated with the numbers of CD3+ T cells in tumor sections from patients with head and neck squamous cell carcinoma, and the expression of galectin-1 and CD3 also served as predictors for the prognosis of such patients [45].

### 3.3. Double-Edged Sword Role of Galectin-9 in Tumors

Unlike other galectins, galectin-9 both promotes and inhibits tumor activity, depending on its interactions with its ligands on T cells, antigen-presenting cells or tumor cells. Previous studies have demonstrated that Epstein-Barr virus-infected nasopharyngeal carcinoma cells (NPCs) release exosomes containing high amounts of galectin-9, which is able to induce TIM-3-expressing Th1 cell apoptosis and subsequently helps the tumor escape immune recognition [1]. The role of galectin-9 in the regulation of the immune response in the tumor microenvironment has been investigated further in patients with recurrent nasopharyngeal carcinoma. Such patients displayed increased galectin-9-positive tumor cells and FOXP3^+^ lymphocytes, whereas the TIM-3^+^ lymphocytes were decreased in the tumor microenvironment compared with primary NPCs, suggesting that the galectin-9–TIM-3 pathway mediates immune escape by NPCs [46]. This pathway also downregulates the antitumor response in hepatitis B virus-associated HCCs. Interestingly, IFN-γ produced by tumor-infiltrating lymphocytes induces galectin-9 production by Küpffer cells. Moreover, increased numbers of TIM-3^+^ T cells in tumors were inversely associated with patient survival, and blockade of the galectin-9–TIM-3 pathway promoted T cell proliferation and secretion of cytokines [47,48]. Besides, galectin-9 also interacts with dectin-1 expressed on macrophages and promotes tolerogenic macrophage programing in the microenvironment of pancreatic ductal adenocarcinoma [36]. These findings suggest that galectin-9 derived from the tumor microenvironment could attenuate antitumor effects and that blockage of this pathway could be a therapeutic target in further clinical applications. In contrast to the tumor-friendly role of galectin-9, results obtained from mouse models in vivo and in vitro indicated that galectin-9 can promote tumor cell apoptosis, including in chronic myelogenous leukemia cells [49], malignant melanomas [50,51], gallbladder carcinomas [52], HCCs [53], cholangiocarcinomas [54] and gastric cancer cells [55]. The mechanism of this galectin-9-mediated cancer cell apoptosis is likely linked to the carbohydrate-recognition function because administration of lactose blocked its proapoptotic effect. Besides, this effect might not act through the activation of the immune system, and the detailed mechanisms—for example, the involvement of endoplasmic reticulum stress-induced apoptosis and cellular receptors—need to be investigated further [56].

## 4. Targeting Galectins or their Ligands in Preclinical and Clinical Trials 

As discussed above, galectins have multiple immunomodulatory effects and can be further applied to develop therapeutic strategies against autoimmunity, graft rejection and tumors. Besides, galectins also have other biological functions including regulating cell migration, adhesion and signal transduction. Given the complexity of the biological functions of galectins, increased levels of galectins in tumors have been reported to promote their growth or interfere with tumor therapies. These mechanisms include interfering with drug efficacy/delivery or dampening the antitumor effect of immune cells.

### 4.1. Galectins Interfere with Chemotherapy against Tumors

The multidrug resistance (MDR) phenotype is a major issue in the development of toxic chemotherapy for treating cancers. Cancer cells develop several mechanisms to combat anticancer drugs, including decreased drug uptake, increased efflux via ATP-binding cassette (ABC) transporters, and increased drug metabolism and/or resistance to drug-induced apoptosis [57]. Previous reports have demonstrated that the chemotherapy drugs adriamycin and imatinib upregulate galectin-1 expression in chronic myelogenous leukemia cells. Galectin-1 confers drug resistance via inducing the expression of MDR protein 1, which in turn helps tumor cells to pump out cytotoxic drugs [58]. Likewise, the expression level of galectin-3 was increased significantly in the sera of patients with various cancers and mediates MDR. Galectin-3 interacted with Na^+^/K^+^-ATPase and P-glycoprotein and enhanced ATPase activity, eventually leading to decreases in doxorubicin-induced cell death [59]. Moreover, in a preclinical mouse model of non-Hodgkin lymphoma using anti-CD20 target therapy, the expression of galectin-1 ablated antibody-dependent lymphoma phagocytosis in vitro and lymphoma cell sensitivity to CD20 immunotherapy in vivo. In addition, biopsies or blood samples from patients with Burkitt lymphoma, chronic lymphoid leukemia, diffuse large B-cell lymphoma, follicular lymphoma, hairy cell leukemia and mantle cell lymphoma displayed increased expression levels of galectin-1, suggesting that this confers resistance to anti-CD20 immunotherapy in humans [60].

In 11 clinical trials (updated to November 2017) registered by the United States National Institutes of Health (https://clinicaltrials.gov/, access on 30 November 2017), there are five using galectin inhibitors (e.g., GM-CT-01, and the Davanat^®^ carbohydrate polymer) combined with chemotherapy drugs (5-fluorouracil) against solid tumors. In these trials, investigators have initiated multicenter phase I or II trials targeting various tumors including colorectal, lung, breast, head and neck, prostate, bile duct and gall bladder cancers, metastatic melanomas and diffuse large B-cell lymphomas. However, only one trial has been completed (Phase I trial, NCT00054977) and the others are neither withdrawn nor terminated, suggesting that not all galectin inhibitors are effective, and that efficacy might also depend on protocol design and galectin expression profiles in each individual (Table 2).

### 4.2. Immunotherapy Combined with Galectin Inhibition

Immunotherapy using monoclonal antibodies blocking immune checkpoint molecules has shown promising progress. However, to increase overall responsiveness, several investigators started to combine these with galectin inhibitors to enhance the therapeutic effect. Until November 2017, two clinical trials have been reported using the galectin-3 inhibitor DG-MD-02 along with ipilimumab (anti-CTLA-4) or pembrolizumab (anti-PD-1) to treat patients diagnosed with melanomas, non-small cell lung cancers, and squamous cell head and neck cancers (Table 2). Excitingly, one of these trials showed that the combination of pembrolizumab with the galectin-3 inhibitor GR-MD-02 gave promising early results in the treatment of patients with advanced melanomas in a phase Ib clinical trial. This mechanistic study showed that clinical responders to this combination might have reduced the numbers of myeloid-derived suppressor cells following treatment. Although this trial showed positive results, the detailed mechanisms are unclear and further clinical trials need to be conducted to demonstrate the efficacy of this approach and to evaluate any possible adverse effects. Despite the use of anti-PD-1 and anti-CTLA-4 antibodies that are effective clinically and the various combination therapies being conducted in clinical trials, several investigators are still working on using other immune checkpoint molecules as targets to enhance the antitumor functions of T cells. The galectin-9 binding partner, TIM-3, which is a negative regulator of T cells is now being used as a novel target in tumor immunotherapy [61,62]. Until November 2017, investigators had conducted three human clinical trials using an anti-TIM-3 monoclonal antibody combined with either anti-PD-1 or anti-PD-L1 antibodies in advanced solid tumors (Table 2). Although these clinical trials are still in their early stages, focusing on characterizing the safety, tolerability, pharmacokinetics, pharmacodynamics and antitumor activity, and blocking multiple negative regulators on T cells might unleash their antitumor effects and eventually help to control tumors.

## 5. Conclusions

Here we have summarized the roles of galectins in human cancer biology and focused on targeting the inhibition of these molecules in ongoing clinical trials. The functions of galectins have been investigated extensively in many fields including cancer cell biology, immunology and infectious diseases. In the earlier studies, researchers have put a lot of effort focus on the roles of galectins in tumor growth/metastasis and correlated the expression level with the prognosis of patients. However, the detailed mechanisms are still unclear, for example, galectin−tumor interaction or reciprocal interaction among galectins, tumor cells and immune cells have not been well examined. Galectin-1, -3 and -9 are three well-investigated galectins which can modulate tumor cells growth and regulate immune cells. To date, these galectins bind a panel of receptors on T cells which inhibit the functions of T cells (Table 1). Thus, tumor cell-derived galectins may not only regulate the growth of tumor cells but also dampen T cell responses. Moreover, galectins may bind to secreted cytokines or chemokines which interfere with immune cell communication [15]. Data obtained from in vitro cell culture or murine xenograft models are significant and informative; however, the results cannot yet be translated directly into clinical settings. Besides, the widely varying expression pattern of galectins and heterogeneity of tumors contribute to difficulties in these studies.

Among the clinical trials using galectin inhibitors, it seems that combined with immune intervention they showed positive results, suggesting that in the well-established tumor, the main function of the increased galectins is to interfere the anti-tumor effects of T cells. Therefore, it is reasonable to believe that using combination therapy (galectin inhibitors, chemotherapy and immune checkpoint blockades) may show great improvements in tumor therapy. However, the protocol (dosage, intervention strategy and side effects) for these clinical trials needs to be further investigated.

Given the complexity of the function of galectins, future works that aim to clarify the effects of galectin in tumors may focus on the following points: to analyze galectin profiles in different clinical samples systematically and to correlate these profiles with tumor stages; to characterize T cell phenotypes of patients with high galectin expression level in tumors, especially for the tumor-infiltrating lymphocytes; to explore the underline mechanism of the altered galectin expression in tumor growth and metastasis, for example, virus−infection−induced galectin overexpression in company with tumorigenesis. Altogether, these results might help in designing effective therapeutic approaches.

## Figures and Tables

**Table 1:** Galectin-1, -3, -9 and their binding partners in the regulation of immune responses and tumor biology.

	Ligand	Targeted Cells	Biological Function	Refs.
Galectin-1	CD2, CD3, CD7, CD43 and CD45	T cells	Apoptosis	[21,22]
	TCR	T cells	Signal transduction	[23]
	Neuropilin 1	Endothelial cells	Cell migration	[24]
	Pre-BCR	Pre-B cells	Signal transduction Cell maturation	[25,26]
	α4 integrins	Pre-B cells	Signal transduction Cell maturation	[25,26]
	H-Ras	Endometrial cancer cells	Membrane anchorage Cell transformation	[27]
	α5β1- integrins	Epithelial cancer cells	Epithelial integrity	[28]
Galectin-3	CD7, CD29, CD45, CD71	T cells	Apoptosis	[29,30]
	TCR	T cells	Signal transduction	[31]
	Alix	T cells	Signal transduction	[32]
	MUC1	Epithelial cancer cells	Signal transduction	[13]
	K-Ras	Breast carcinoma cells	Enhanced K-Ras stability	[33]
	TTF-1	Thyroid cancer cells	Tumor progression	[34]
	Mac-2BP	Melanoma cells	Cell-cell adhesion	[35]
Galectin-9	Dectin-1	Macrophages	Tolerogenic macrophage programming and adaptive immune suppression	[36]
	TIM-3	Dendritic cells, monocytes	Maturation and cytokine production	[37,38]
	TIM-3	T cells	Apoptosis	[39]
	4-1BB	T cells	Signal transduction	[40]
	CD40	T cells	Inducing cell death and suppressing proliferation	[41]

**Table 2:** Targeting galectins and their ligands in clinical trials.

Status	Condition	Intervention	Phase	Sponsors and Collaborators
Completed	Colorectal, lung, breast, head and neck, and prostate cancers	GM-CT-01 (galectin-3 inhibitor) combined with 5-fluorouracil	Phase I NCT00054977	Galectin Therapeutics Inc.
Withdrawn	Cancers of the bile duct and gallbladder	GM-CT-01 (galectin-3 inhibitor) combined with 5-fluorouracil	Phase II NCT00386516	Galectin Therapeutics Inc.
Terminated	Colorectal cancer	GM-CT-01 (galectin-3 inhibitor) combined with 5-fluorouracil, leukovorin, bevacizumab	Phase II NCT00388700	Galectin Therapeutics Inc.
Unknown status	Metastatic melanoma	Tumor peptide vaccination combined with GM-CT-01 (galectin-3 inhibitor)	Phase I/II NCT01723813	Cliniques Universitaires Saint-Luc Université Catholique de Louvain
Unknown status	Solid tumors	OTX008 inhibitor of galectin-1 expression	Phase I NCT01724320	Oncoethix GmbH
Withdrawn	Diffuse large B-cell lymphoma	GCS-100 (galectin-3 inhibitor)	Phase I/II NCT00776802	La Jolla Pharmaceutical Co. investigators
Recruiting	Metastatic melanoma	GR-MD-02 (galectin-3 inhibitor) combined with ipilimumab (anti-CTLA-4)	Phase IB NCT02117362	Providence Health & Services, Providence Cancer Center, Earle A. Chiles Research Institute; Galectin Therapeutics Inc.
Recruiting	Melanoma, non-small cell lung cancer, and squamous cell head and neck cancers	GR-MD-02 combined with pembrolizumab (anti-PD-1; keytruda)	Phase IB NCT02575404	Galectin Therapeutics Inc.; Providence Health & Services
Recruiting	Advanced or metastatic solid tumors	TSR-022 (anti-TIM-3) combined with anti-PD-1 antibody	Phase I NCT02817633	Tesaro, Inc.
Recruiting	Solid tumors	LY3321367 (anti-TIM-3) combined with LY3300054 (anti-PD-L1)	Phase I NCT03099109	Eli Lilly and Co.
Recruiting	Advanced Malignancies	MBG453 (anti-TIM-3 ) combined with PDR001 (anti-PD-1)	Phase I/II NCT02608268	Novartis Pharmaceuticals
Recruiting	relapsed/refractory acute myeloid leukemia	MBG453 (anti-TIM-3 ) combined with PDR001 (anti-PD-1) and/or decitabine (5-aza-2′-deoxycytidine)	Phase I NCT03066648	Novartis Pharmaceuticals
